# Potential Human Health Risk by Metal(loid)s, ^234,238^U and ^210^Po due to Consumption of Fish from the “Luis L. Leon” Reservoir (Northern México)

**DOI:** 10.3390/ijerph110706612

**Published:** 2014-06-25

**Authors:** Mayra Y. Luna-Porres, Marco A. Rodríguez-Villa, Eduardo F. Herrera-Peraza, Marusia Renteria-Villalobos, María E. Montero-Cabrera

**Affiliations:** 1 Department of Renewable Energy and Environmental Protection, Advanced Materials Research Center (CIMAV), Miguel de Cervantes 120, Chihuahua, 31109, Mexico; E-Mails: mayra.luna@cimav.edu.mx (M.Y.L.-P.); eduardo.herrera@cimav.edu.mx (E.F.H.-P.); 2 Department of Materials Science, Autonomous University of Ciudad Juarez, Ave. del Charro 450 Norte, Cd. Juárez 32310, Mexico; E-Mail: al118805@alumnos.uacj.mx; 3 Department of Natural Resources, Autonomous University of Chihuahua, Periferico Francisco R. Almada, Chihuahua, 33820, Mexico; E-Mail: mrenteria@uach.mx

**Keywords:** bioaccumulation, metal(loid)s, fish, *Lepomis cyanellus*, *Cyprinus carpio*, *Ictalurus furcatus*, uranium, polonium, Chihuahua

## Abstract

Concentrations of As, Cu, Fe, Hg, Pb and Zn and activity concentrations from ^234,238^U and ^210^Po in water, fillet, liver and gills were determined in three stocked fish species from the Luis L. Leon reservoir, located in Northern Mexico. The considered species were *Lepomis cyanellus*, *Cyprinus carpio* and *Ictalurus furcatus*. ^238^U and ^234^U activity concentration (AC) in fillet samples showed values of 0.007–0.014 and 0.01–0.02 Bq∙kg^−1^ wet weight (ww), respectively. Liver samples for *L. cyanellus*, *C. carpio* and *I. furcatus* present ^210^Po AC of 1.16–3.26, 0.70–1.13 and 0.93–1.37 Bq∙kg^−1^ ww. Arsenic, mercury and lead concentration intervals in fillet samples were 0.13–0.39, 0.005–0.126 and 0.009–0.08 mg∙kg^−1^ ww, respectively, while in gill samples they were 0.11–0.43, 0.002–0.039 and 0.02–0.26 mg∙kg^−1^ ww. The elemental Bioaccumulation Factor (BAF) for fish tissues with respect to their concentrations in water was determined. *L. cyanellus* showed the highest BAF values for As and total U, being BAF_As_ = 37 and 40 L∙kg^−1^ in fillet and gills, respectively, and BAF_U total_ = 1.5 L∙kg^−1^ in fillet. *I. furcatus* showed the highest BAF values for Hg and Pb, being BAF_Hg_ = 40 and 13 L∙kg^−1^ in fillet and gills, and BAF_Pb_ = 6.5 and 22 L∙kg^−1^ in fillet and gills, respectively. Some metal(loid) concentrations are slightly higher than European regulations for fish fillets. The difference in concentrations of metal(loid)s in fillet among the studied species is probably due to their differences in diet and habitat.

## 1. Introduction

Historically, levels of metal(loid)s in aquatic ecosystems have been increasing, due to mining, industrial and agricultural activities [1,2,3,4,5,6,7]. Metal(loid)s in the aquatic environment are bioaccumulative, not biodegradable and may be incorporated into the food chain. The consumption of fish loaded with metal(loid)s may impact human health. For example, arsenic has been recognized as a very hazardous element pollutant. Skin diseases like hyperpigmentation, keratosis, and possible vascular complications, as well as different types of cancer have been attributed to their ingestion or inhalation [8]. Lead is a cumulative pollutant that affects multiple body systems, including the neurologic, hematologic, gastrointestinal, cardiovascular, and renal systems [9]. Intake of uranium has toxic effects, particularly in the urinary system [10].

Fish are good bioindicators of pollution in the aquatic environment, because they accumulate metal(loid)s in a manner depending on their position in the trophic levels and their feeding habits. In addition, fish are easily sampled and they are of different sizes and ages [11,12]. As a consequence of pollution, regulated intake of fish has been suggested [13]. 

Metal(loid)s content in fish and water has been the subject of many recent studies [12,14,15,16,17,18]. The importance of studying radionuclide concentrations in fish has been emphasized as well. The oxidative stress generated in fish exposed to uranium is probably a result of the stimulation of reactive oxygen species production in the course of redox reactions, causing damage to tissues by alteration of nuclear acids, proteins, lipids or carbohydrates [19,20]. ^210^Po is considered to be one of the most toxic naturally occurring radionuclides [21]. Moreover, high uranium and ^210^Po concentrations have been found in freshwater fish tissues from reservoirs polluted by uraniferous tailings [22,23,24].

The concentration of metal(loid)s in gills reflects the concentration of these elements in the water where fish live. In addition, the liver function in the body of animals results in the accumulation of toxins in their tissues, including metal(loid)s [25]. Thus liver and gills in fish are often recommended as environmental indicator organs of water pollution [26]. 

Intake of radionuclides and metal(loid)s by fish depends on their bioavailability, which in turn depends on their ionic species and on possible chemical compounds interacting with external organs or being ingested. Concentration of metal(loid)s vary according to their speciation during exposure, degree of biomagnification and target organs [27,28]. 

**Figure 1:**
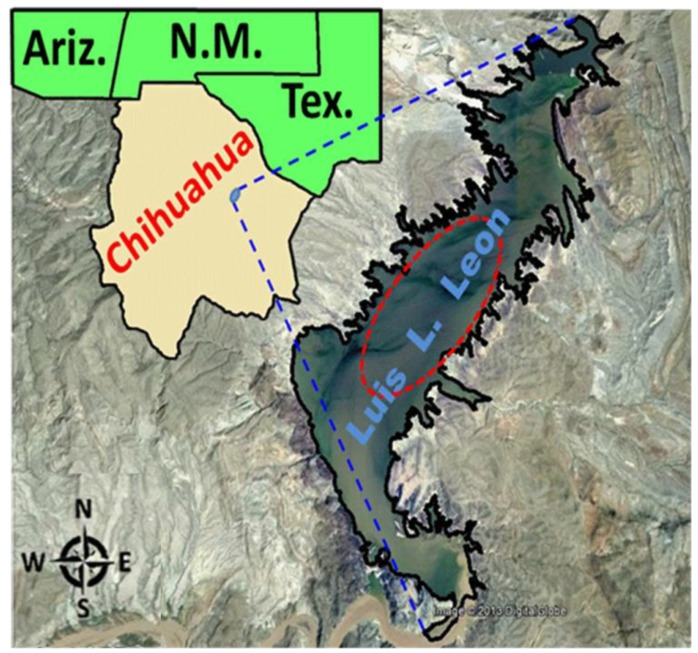
Luis L. Leon reservoir in Chihuahua State. The location of fish sampling (ellipse) within the reservoir is represented.

The Conchos River is one of the most important water sources in Northern Mexico and the main surface waterway in the arid state of Chihuahua. The Luis L. Leon reservoir is the last major reservoir before the Conchos River enters the Rio Grande at the Texas-Chihuahua border (see Figure 1 below). This reservoir provides flood control and it is a major source of irrigation for pasture and cropland, as well as a location for recreational and commercial fishing. Furthermore, the Water Treaty of 1944 for the “Utilization of Waters of the Colorado and Tijuana Rivers and of the Rio Grande” established the amount of water that United States and Mexico should share from different basins. The amount and quality of water from Conchos River tributary to Rio Grande is controlled essentially by the Luis L Leon reservoir [29].

Many uranium deposits are reported in the State of Chihuahua [30], and as a result, an environmental radiological surveillance program has been established. Through various studies conducted in the Sacramento-Chuviscar River basin, high activity concentrations (AC) of radionuclides in surface water, groundwater, sediments and biota have been found [31,32,33]. Based on these studies, we have identified the need to know if uranium and other radioactive isotopes may reach the reservoir Luis L. Leon, after transport or leaching from Chihuahua Sacramento Valley to the Conchos River. To our best knowledge, radioactivity in water, sediments or fish has not been reported for this reservoir. 

In the last decade, some investigations on water quality and trace element contaminants (not including radioisotopes) in several of the main reservoirs and rivers in the State of Chihuahua, as well as in sediments and fish were conducted [34,35,36,37]. In some cases, high concentrations of contaminants in the three compartments were reported.

The aim of this study is to investigate the incorporation of ^234,238^U, ^210^Po and metal(loid)s by three species of stocked fish (*Lepomis cyanellus*, *Cyprinus carpio* and *Ictalurus furcatus*) captured from Luis L. Leon reservoir in two different seasons. These species are of interest because of their economic importance in the region and their consumption by the local population. The study followed these steps: (1) the determination of ^234,238^U and metal(loid) concentrations in water and fillet samples, metal(loid) concentrations in gills, and ^210^Po in liver of the selected fish species; (2) the calculation of the contaminants incorporation in fish using the Bioaccumulation Factor (BAF) relative to water content; (3) the assessment of the potential effects on human health due to fish consumption, by comparison of results with European regulations and by estimation of the theoretical dose by metal(loid)s and uranium ingestion.

## 2. Materials and Methods

### 2.1. Study Area

The Luis L. Leon reservoir (Figure 1, UTM coordinates: 471689/3204730) is located approximately 90 km Northeast of Chihuahua City. This reservoir, with a capacity of 853.94 million m^3 ^was built in 1968. The main exposed rocks in the region are limestone, igneous intrusive and volcanic whose ages range is from the Upper Jurassic to the Recent [38]. In this section, the Rio Conchos water flows from South to North towards the Rio Grande.

### 2.2. Sampling

Samples of water and three species of fish *Lepomis cyanellus, Cyprinus carpio* and *Ictalurus furcatus* (commonly named bluegill sunfish, common carp and catfish, respectively) were collected in 2011 and 2012. Sampling campaigns were grouped into Winter-Spring (W-S, from November to April) and Summer-Autumn (Su-A, from May to October). Samples of adult fish specimens were collected by local fishermen. 

As fish usually move across the water column to depths of about 10 m, water samples were collected at the fishing sites at 0.1 and 10 m depths in brand new 5 L capacity polyethylene containers that were pre-washed with distilled water. Parameters like pH, temperature, and total dissolved solids (calibrated Waterproof Oakton pH Tester 30 and TDSs-Oakton TDS Tester 10w), were measured *in situ*. All collected samples were kept on ice until reaching the laboratory, and then in a refrigerator. 

#### Sample Preparation

Fish samples were vigorously washed with deionized water to remove external contamination. The fish samples were dissected to separate the fillet, gills and liver which were then analyzed, as described below. Subsequently, these tissues were dried, homogenized, and crushed with a grinder for further analysis [16]. Water samples were acidified with analytical grade HNO_3_ to 10% until further analysis.

The preparation and analysis of fish gill and fillet samples was carried out individually for all fish species. However, the liver samples obtained did not provide sufficient tissue for analysis. Then, to obtain enough amounts of liver tissue for polonium determination, collection of every six samples were pooled to produce three samples for the liver of *Lepomis cyanellus, Cyprinus carpio* and *Ictalurus furcatus.*

### 2.3. Analytical Methods and Measurement Techniques

^234,238^U activity concentration was determined using a photon/electron rejecting alpha liquid scintillation (PERALS) liquid scintillation spectrometer with alpha-beta separation from ORDELA (Oak Ridge, TN, USA) [39]. ^210^Po activity concentration was determined using alpha spectrometry with surface barrier silicon PIPS detectors from Canberra (Meriden, CT, USA). Concentrations of As, Cu, Fe, Hg, Pb and Zn were determined by Inductive coupled plasma optical emission spectrometry (ICP-OES) with an iCAP series 6000 from Thermo Scientific (Waltham, MA, USA).

All reagents were of analytical grade and only deionized water was used; all laboratory equipment and containers were washed in 10% HNO_3_ solution for 20 min and rinsed with deionized water prior to each use. Blank samples were processed to check contamination from materials or used reagents.

For water quality analysis, cation contents was determined by the method NMX-AA-051-SCFI-2001 [40], with a GBC model Avanta Sigma (Waltham, MA, USA) atomic absorption spectrophotometer, calibrated with certified reference materials. Nitrates, sulfates and hardness were determined by HACH method 8171, 8051 and 8204 [41] respectively, and chlorides by the NMX-AA-073-SCFI-2001 method [42]. Alkalinity was determined using the NMX-AA-036-SCFI-2001 method [43].

#### 2.3.1. Radiochemical Method

Fillets and water samples were subjected to pre-concentration procedures. Fillet samples were subject to drying and calcination for 12 and 24 h at 56 °C and 600 °C, respectively. Pre-concentration of water samples was obtained by evaporation from 5 to 0.2 L. First, a known amount (approximately 0.5 mL) of ^232^U (NIST SMR432 B, specific activity 0.129 ± 0.001 Bq∙L^−1^) as a standard yield tracer was added to fish fillet and water samples. Then, samples were subject to acid attack on hot plate digestion (8 M nitric acid and hydrogen peroxide). After that, uranium was extracted using URAEX scintillation cocktail [16,44]. Finally, in order to improve energy resolution, the sample in the scintillation vial was bubbled with a gas mixture of argon and toluene (for extraction of dissolved oxygen) [39]. The chemical yield of the analysis ranged from 88% to 96%.

For accuracy determination in ^234,238^U activity measurements, a natural uranium reference material (high-purity standard 100064) was added to some fish samples before they were subjected to the analytical procedure. Results for ^238^U and ^234^U were 0.598 and 0.573 Bq, respectively, with relative uncertainty of 3%, while those reported from the reference material were 0.617 ± 0.002 Bq to ^238^U and 0.599 ± 0.002 Bq to ^234^U. The calculated limit of detection was 0.0002 Bq∙kg^−1^ [45].

Liver samples were subjected only to drying without calcination (avoiding thus polonium losses). First a standard yield tracer ^209^Po (specific activity = 200.7 ± 3.8 mBq∙mL^−1^) was added to samples in a known amount (approximately 0.5 mL) for chemical recovery determination of the analysis. Then, the samples were digested using 10 mL of concentrated nitric acid during 12 h at 80 °C. After evaporation to dryness, the residues were dissolved in HCl. Finally, autodeposition of polonium was done in a solution of ascorbic acid in 1.5 M HCl. The solution was heated to 80 °C for 4 h and Po was spontaneously plated onto a rotating silver disc. Details about these procedures were described in [16]. 

#### 2.3.2. Metal(loid)s Analysis

To follow with the elemental analysis, fish fillets, gills and water samples were separately subject to acid attack on a hot plate (in a closed system). At first, all samples were added with gold, to amalgamate mercury and avoid potential losses. The samples were digested in concentrated nitric-hydrochloric acid for about 6 h at 60 °C, until the orange fume of nitric acid completely disappeared. Then, about 3 mL of peroxide were added. Finally, each sample was evaporated to about 4 mL, cooled, diluted up to 10 mL with distilled water and filtered with Whatman filter 2. The gills were filtered with Whatman filter 42 because of their higher than fillet fat content, to ensure safe performance of the spectrometer sample introduction system [37,46,47].

The calibration curve of the ICP-OES was obtained using a standard solution of QCS-27 (Quality Control Standard 27) in 4% HNO_3_ + Tr HF (High-purity standards 1130517) [48]. The accuracy and precision of the method (Table 1) was determined by analyzing the certified reference material DOLT-4. The detection limits were (in mg∙kg^−1^ ww): As = 0.0001, Cu = 0.0004, Fe = 0.0004, Hg = 0.0001, Pb = 0.0001 and Zn = 0.0002.

**Table 1:** Accuracy (given as recovery) and precision (given as standard deviation) of standard DOLT-4, analyzed by the procedure employed in fish metal(loid)s analysis.

Element	DOLT-4 concentration (mg∙kg^−1^ dw ± SD)		Recovery (%)
	Certified	Measured	
As	9.66 ± 0.62	8.7 ± 0.6	90
Cu	31.2 ± 1.1	28.4 ± 0.6	91
Fe	1833 ± 75	1686 ± 40	92
Pb	0.16 ± 0.04	0.15 ± 0.03	93
Hg	2.58 ± 0.22	2.76 ± 0.13	107
Zn	116 ± 6	113 ± 8	97

Notes: dw: dry weight; SD = standard deviation; DOLT-4 *=* Dogfish Liver Certified Reference Material for Trace Metals Analysis-National Research Council of Canada.

### 2.4. Bioacumulation Factor

Bioaccumulation is defined as the net result of the absorption, distribution, and elimination of a substance in any organism after exposure [24,49,50,51,52]. 

The incorporation of contaminants in fish was calculated by a Bioaccumulation Factor (BAF), relative to the contaminant content in water, which is given by Equation (1) in L∙kg^−1^:

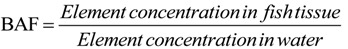
(1)
BAF was calculated for gills and fillet of the three fish species. 

### 2.5. Estimates of Theoretical Intake and Effective Dose for Metal(loid)s in Fish

Theoretical estimations of contaminant intake and effective dose by ingestion of uranium have been calculated on the basis of concentrations in fillet for each fish species examined in this study. 

#### 2.5.1. Intake Estimation

The metal(loid)s intake estimation was performed in two ways: Estimated Daily Intake (EDI), for essential elements (such as Cu, Fe and Zn) and Estimated Weekly Intake (EWI), for non-essential elements [53]. 

The intake was estimated by the Equation (2). The calculation is performed on a daily or weekly basis by changing the period over which the average is taken. EDI is given in µg∙day^−1^ and EWI in µg∙week^−1^:


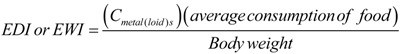
(2)

The calculations were made on the basis of average fish consumption: 0.02 kg per person per day^−1^ and 0.14 kg per person per week^−1^ [54], assuming a person body weight of 70 kg [55].

#### 2.5.2. Effective Dose Estimation

Annual effective dose (H) is a useful concept that enables the radiation doses from different radionuclides and from different types and sources of radioactivity to be added. It is based on the risk of radiation induced health effects as defined by the International Commission on Radiological Protection (ICRP) [5,56]. The effective dose (H) by U ingestion in fish was calculated by Equation (3) (in µSv∙year^−1^):

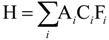
(3)
where A*_i_*, C*_i_* and F*_i_* denotes the activity concentration of the radionuclide of interest (Bq∙kg^−1^), the consumption rate (kg∙year^−1^) and the conversion coefficient for the ingestion of the i-radionuclide in tissue. For adults so called *member of the public*, the recommended dose conversion coefficients are: ^238^U = 4.5 × 10^−2^ µSv∙Bq^−1^ and ^234^U = 4.9 × 10^−2^ µSv∙Bq^−1^, respectively [57]. Equation (3) calculations were made on the basis of an average fish consumption of 7.3 kg per year. 

### 2.6. Statistical Analysis

All results of concentration of metal(loid)s and radioactivity in fillet and gills, as well as water, were analyzed statistically using the Minitab 16 Statistical Software (Minitab Inc., State college, PA, USA). A multi-way MANOVA approach, which reveals whether there is significant differences (p <0.05), was performed to address the factorial effects and interaction effects of: (1) sampling campaign and fish species in uranium and metal(loid) concentrations in the fillet; (2) sampling campaigns, fish species and tissue (gills *vs.* fillet) in metal(loid) concentrations found in the fish; (3) sampling campaign and sampling depth in concentrations of uranium, metal(loid)s, as well as in physicochemical parameters. As significant effects of factors were found for a variable, ANOVA followed by the Tukey *post-hoc* test was then run to assign differences to treatment levels. The Pearson correlation coefficient was also calculated to study the relationship between different concentrations of metal(loid)s and uranium, for each matrix studied (fillet, gills and water). In the case of biological specimens, they are classified by species under study (*C. carpio*, *I. furcatus*, *L. cyanellus*). The results of statistical analyzes are presented in the Appendix, Tables A3 and A4, for those cases where significant differences (*p* < 0.05) exist.

## 3. Results and Discussion

### 3.1. General Information of Analyzed Species

Table 2 provides the common and scientific names, the length and weight ranges of the stocked fish species caught at Luis L. Leon reservoir, as well as a brief description of their diet and habitat. The population showed a linear length increasing with weight (see Equation in Table 2) for the size ranges of the three species presented (*R*^2^ ≥ 0.9, see Table 2).

### 3.2. ^234,238^U and ^210^Po Activity Concentration

Table 3 presents the geometric mean values of the uranium activity concentration in fish fillets (wet weight—ww), in water samples and also the BAF in fillets from Equation (1). BAF was calculated using the geometric mean of the uranium isotopes AC in fillets from all samples (*n* = 24) for each species and the average AC from water taken at both different depths. These geometric mean values were calculated taking results from all samples, indistinctly of season, for better integral assessment of bioaccumulation; the mean AC in water was used after checking that no significant differences between the measured values for the two depths were observed; see below for details. For instance, *Lepomis cyanellus*: 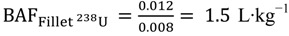. From results of Table 3, uranium activity concentrations present in fish fillet are low, in comparison with both the reference values established by UNSCEAR 2008 [57], and the values of 0.14–2.6 and 0.6–4.5 Bq∙kg^−1^ (dry weight) for ^238^U and ^234^U, respectively, reported in samples of species *Cichlasoma labridens* in San Marcos Dam (Chihuahua) [33]. The uranium concentrations found in this study are also lower than those presented in other publications related with uranium mine activities [58,59]. The activity ratios ^234^U/^238^U were 1.3–1.5 in fillet samples and 1.1–2.7 in water. It is noteworthy that this slight disequilibrium was expected, because it is known that ^234^U is more soluble in water than ^238^U and it is well known that isotope disequilibria of ^234^U/^238^U in natural waters can occur due to alpha recoil effects [22]. Studies developed in San Marcos Dam have reported a greater radioactive disequilibrium between ^234^U and ^238^U than the ones reported in the present work. Renteria-Villalobos *et al.* [33] reported significantly higher radioactive disequilibrium in fillet of species *Cichlasoma labridens*, ranging from 0.9–4.5. Also Burillo-Montufar *et al.* [60] found activity ratios in water samples ranging from 1.1 to 1.9. However, the differences in isotopic activity concentration may be attributed to the geological environment where San Marcos Dam is placed.

That dam stores water from San Marcos River, which runs close to Victorino and San Marcos uranium deposits, located in that region [61]. On the contrary, the environment around the Luis L. Leon reservoir is essentially formed by limestone. Some geothermal waters may have influence [38]. The municipality of Aldama is known for the presence of gypsum deposits [62] and by the outcrop of gypsum surface sediments after these geothermal events in the vicinity of Chuviscar River and the Rio Conchos.

**Table 2:** General information and relationships between weight in kg (W) and total length in cm (L) of the fish species caught from the Luis L. Leon reservoir [63].

Scientific Name	Common Name	Diet	Habitat	*n*	L. Range	W. Range	Equation	*R*^2^ Value
*Cyprinus carpio*	Common carp	Insects, Plant, Algae	Cloudy or stagnant waterin areas with less than 10 m deep	24	30–45 (36 ± 3.9)	0.10–0.30 (0.16 ± 0.04)	*L* = 22.29 + 83.44*W*	0.90
*Lepomis cyanellus*	Bluegill	Insects, Small fish, Shellfish	Shallow areas with sand, gravel and vegetation	24	31–48 (36 ± 4.5)	0.11–0.23 (0.16 ± 0.03)	*L* = 16.63 + 124.1*W*	0.92
*Ictalurus furcatus*	Catfish	Fish, Insect larvae and mollusks Detritus	Environments with braking currents on sand or rock bottom	24	30–49 (39 ± 5.5 )	0.11–0.31 (0.20 ± 0.06)	*L* = 21.48 + 89.07*W*	0.92

Notes: *n* = sample size; *R*^2^ = coefficient of determination.

**Table 3:** Geometric mean values of uranium isotope AC in water (Bq∙L^−1^), fish fillets (Bq∙kg^−1^ ww), activity ratio and BAF (L∙kg^−1^) and ^210^Po AC in Bq∙kg^−1^ ww in liver for fish sampled at Luis L. Leon reservoir.

Sample	*n*	Tissue	Activity concentrations				
			^238^U	^234^U	U Total	^234^U/^238^U	^210^Po
*Cyprinus carpio*	24	Fillet	*0.008* (0.003–0.025)	*0.012* (0.004–0.042)	*0.020* (0.007–0.067)	1.5 (1.1–1.6)	--
*Ictalurus furcatus*	24	Fillet	0.011 (0.003–0.034)	0.016 (0.006–0.048)	0.028 (0.01–0.076)	1.5 (1.1–1.6)	--
*Lepomis cyanellus*	24	Fillet	**0.012** (0.004–0.034)	**0.017** (0.005–0.041)	**0.029** (0.008–0.075)	1.4 (1.1–1.5)	--
*Cyprinus carpio*	18	Liver	--	--	--	--	*0.85* (0.7–1.13)
*Ictalurus furcatus*	18	Liver	--	--	--	--	1.13 (0.93–1.37)
*Lepomis cyanellus*	18	Liver	--	--	--	--	**1.73** (1.16–3.26)
Water *	8		0.008 (0.004–0.012)	0.011 (0.006–0.018)	0.02 (0.01–0.03)	1.8 (1.1–2.7)	--
BAF **	24		*0.94* ^1^	*1.0* ^1^	*1.0* ^1^	--	--
	24		1.3 ^2^	1.4 ^2^	1.4 ^2^	--	--
	24		**1.5** ^3^	**1.5** ^3^	**1.5** ^3^	--	--

Geometric means, values in parentheses indicate the minimum and maximum. *n* = sample size. Numbers in bold and italic formats indicate the minimum and maximum geometric mean values. Different numbers indicate the three species fish analyzed: ^1^ *Cyprinus carpio*, ^2^ *Ictalurus furcatus*, ^3^ *Lepomis cyanellus*. * Geometric mean of water samples collected at 0.1 and 10 m depths in the two sampling campaigns; ** BAF calculated from concentration de uranium in fillet.

In the water samples analyzed in this study at two depths, there was a slight difference between the uranium AC. The samples collected at 10 m depth showed 24% higher concentrations than surface water samples. However, there was no statistically significant difference between those uranium activity concentrations (ANOVA, *p* > 0.05). The geometric mean of activity concentrations for fish species depending on sampling campaigns is presented in Table A1 in the Appendix.

There was a slight difference between the uranium AC values obtained for fillet samples at the two sampling campaigns. All fillet samples showed the highest ^234,238^U activity concentrations in Winter-Spring. In addition, the highest ^234,238^U activity concentration occurs in the species *Lepomis cyanellus*. Nevertheless, there were no statistically significant differences between sampling campaigns of Winter-Spring and Summer-Autumn (ANOVA, *p* > 0.05).

*Lepomis cyanellus* showed the highest BAF for uranium activity. In spite of this, no significant differences (ANOVA, *p* > 0.05) were found in the total activity concentration in the three different fish species analyzed. Only ^238^U AC shows statistically significant differences among species (ANOVA, *df* = 2, F = 3.38 and *p* = 0.04, see Appendix, Table A3, for arithmetic mean and *p* values).

Uranium bioaccumulation factor was low for all species, especially if compared with results reported in Carvalho *et al.* and Kraemer and Evans [58,59]. As uranium concentration values in fish and sediments of Luis L Leon reservoir have not ever been reported, there is no information about possible sources of uranium bioaccumulation in fish.

^210^Po activity concentrations in liver samples were 1.16, 1.37 and 3.26 Bq∙kg^−1^ ww for *Lepomis cyanellus*; 0.70, 0.79 and 1.13 Bq∙kg^−1^ ww for *Cyprinus carpio* and 0.93, 1.13 and 1.37 Bq∙kg^−1^ ww for *Ictalurus furcatus*.

Polonium concentration in fish muscle in Syria [64] shows higher concentrations in sea fish than in freshwater fish. Concentrations for ^210^Po in muscle samples were reported as 0.27–27.48 Bq∙kg^−1^ ww and 0.61–3.08 Bq∙kg^−1^ ww for sea and freshwater fish, respectively. These variations in ^210^Po content in species were attributed to differences in metabolism and feeding patterns [64]. Determination of polonium in samples from *Sparus aurata,* reported by Luna *et al.* [16] showed higher AC values in liver than in fillet samples. This behavior is expected because this organ typically bioaccumulates more polonium [65].

### 3.3. Metal(loid)s

Table 4 presents metal(loid) concentrations in ww for fish fillet and gill and water samples, as well as BAF obtained by Equation (1). BAF was calculated using the geometric mean of the metal(loid) concentrations in fillet (*n* = 24) for each species and average concentrations in water samples taken from the two different depths, as in case of BAF for analyzed radioisotopes, see above. Table A2 in the Appendix is displaying the geometric means of metal(loid) concentrations for fish species depending on sampling campaigns.

The ranges of some metal(loid) concentrations in fillet samples shown in Table 4 are similar to those reported by Moreno *et al.* [37] for the *Lepomis macrochirus* and *Cyprinus carpio*, captured at Luis L. Leon reservoir (As = 0.009–0.94 mg∙kg^−1^, Cu = 0.18–6.8 mg∙kg^−1^, Fe = 0.8–15 mg∙kg^−1^, Hg = 0.021–1.2 mg∙kg^−1^, Pb = 0.21–1.8 mg∙kg^−1^, Zn = 2.5–16 mg∙kg^−1^, all given in wet weight). 

**Table 4:** Geometric mean values of metal(loid)s concentration in water (mg∙L^−1^), fish tissues (mg∙kg^−1^ ww) and BAF (L∙kg^−1^) for fillet and gills in fish sampled at Luis L. Leon reservoir.

Sample	*n*	Tissue	Metal(loid)s concentration					
			As	Cu	Fe	Hg	Pb	Zn
*Cyprinus carpio*	24	Fillet	*0.15* (0.03–0.56)	0.18 (0.02–0.46)	4.2 (1–11)	*0.008* (0.0003–0.34)	0.037 (0.0003–0.32)	11.1 (4–19)
	24	Gill	*0.12* (0.02–0.45)	*0.15* (0.06–0.26)	**11.0** (5–19)	*0.004* (0.0003–0.19)	*0.14* (0.018–0.48)	13.5 (15–85)
*Ictalurus furcatus*	24	Fillet	0.15 (0.04–0.28)	*0.14* (0.01–0.36)	**4.4** (0.3–12)	**0.079** (0.0005–0.88)	**0.065** (0.002–1.48)	*8.9* (2–16)
	24	Gill	0.17 (0.01–0.52)	0.2 (0.08–0.4)	10.3 (6–18)	**0.025** (0.0003–0.22)	**0.22** (0.01–0.53)	**14.5** (14–91)
*Lepomis cyanellus*	24	Fillet	**0.29** (0.10–0.65)	**0.22** (0.06–0.62)	*3.2* (1–12)	0.069 (0.0003–1.02)	*0.023* (0.0003–0.28)	**11.4** (4–18)
	24	Gill	**0.31** (0.1–0.7)	**0.23** (0.01–0.58)	*8.3* (5–19)	0.014 (0.0003–0.25)	0.185 (0.011–0.38)	*13.1* (14–81)
Water *	8	--	0.008 (0.003–0.01)	0.014 (0.01–0.06)	0.044 (0.007–0.25)	0.002 (0.001–0.009)	0.009 (0.002–0.1)	0.016 (0.003–0.28)
BAF **	24	Fillet	*19* ^1^	13 ^1^	96 ^1^	*4* ^1^	3.7 ^1^	**594** ^1^
	24	Gill	*15* ^1^	11 ^1^	**249** ^1^	*2* ^1^	*14* ^1^	720 ^1^
	24	Fillet	19 ^2^	10 ^2^	**100** ^2^	**40** ^2^	**6.5** ^2^	*469* ^2^
	24	Gill	22 ^2^	14 ^2^	233 ^2^	**13** ^2^	**22** ^2^	**776** ^2^
	24	Fillet	**37** ^3^	16 ^3^	*73* ^3^	35 ^3^	*2.3* ^3^	554 ^3^
	24	Gill	**40** ^3^	17 ^3^	*188* ^3^	7 ^3^	19 ^3^	*702* ^3^

Geometric means, values in parentheses indicate the minimum and maximum; n = sample size; Numbers in bold and italic formats indicate the minimum and maximum geometric mean values for studied tissues and metal(loid)s; Different numbers indicate the three species fish analyzed: ^1^ *Cyprinus carpio*, ^2^ *Ictalurus furcatus,* ^3^ *Lepomis cyanellus*; * Geometric mean of water samples collected at 0.1 and 10 m depths in the two sampling campaigns; ** BAF calculated from concentrations of metal(loid)s in fillet and gills.

The ranges of some metal(loid) concentrations in gill samples (see Table 4), are also similar to those reported by Moreno *et al.* [37] (As = 0.084–1.67 mg∙kg^−1^, Cu = 0.08–0.58 mg∙kg^−1^, Fe = 9–46 mg∙kg^−1^, Hg = 0.013–0.084 mg∙kg^−1^, Pb = 0.73–6.4 mg∙kg^−1^, Zn = 10.11–189 mg∙kg^−1^, all given in wet weight). However, Moreno *et al.* [37] found higher maximum concentrations for As, Hg and Pb in both tissues (fillet and gills). The decrease of arsenic concentration in the last years at this reservoir, and then the detection of smaller concentrations in fish tissue, may be related to the implementation of measures to prohibit the discharge of waste water into water bodies in the State of Chihuahua.

On the other hand, the National Water Commission of Mexico has reported in 2001 in water analysis in the same Luis L Leon reservoir some metal(loid) concentrations in the water similar to the present results, such as: As = 0.0083–0.03, Cu = 0.002–11.032, Fe = 0.058–0.234, Hg = 0.004, Pb = 0.00095–0.02 and Zn < 0.024, all given in mg∙L^−1^ [66]. Arsenic in water of the reservoir has been reported by Gutierrez *et al.* [35] to be 0.0042 mg∙L^−1^. Mercury in water has been reported by Gutierrez and Borrego [67] at a lower concentration of 0.03 ± 0.05 µg∙L^−1^ in the section of Conchos River that includes Luis L Leon reservoir. Some water sampling points in the reports given by the National Water Commission of Mexico [66] and by Gutierrez *et al.* [35] are close to the sampling area in this study. However, the water sampling points of Gutierrez and Borrego [67], are several kilometers far to the North from those of the present study area. This fact, and the difference in dates, could explain the difference in the Hg concentration in water for the same reservoir: the water flows in this reservoir from the South to the North, and the vegetation and accidents at the bottom of the lake in some extend may filter contaminants from the water.

The geometric mean of physicochemical parameters of water samples collected at 0.1 and 10 m depths in the two sampling campaigns are: pH = 8 ± 0.3, Temperature = 19 ± 2 °C, Total Disolved Solids = 171 ± 59 ppm, Conductivity = 1384 ± 80 µs∙cm^−1^. Meanwhile, concentration of majors ions are: Total hardness (CaCO_3_) = 213 ± 39 mg∙L^−1^, Mg^+2^ = 13 ± 1 mg∙L^−1^, NO_3_**^−^** = 0.88 ± 0.6 mg∙L^−1^, SO_4_**^−^**^2^ = 10 ± 7 mg∙L^−1^. According to pH, the water is characterized as slightly alkaline. 

Although fish specimens of the three species did not differ much in their length and weight (Table 2), their fillet showed variable metal(loid) concentrations. Zn and Fe concentrations are high and similar for all species. Besides, concentrations of metal(loid)s vary as follows: *Lepomis cyanellus*: As > Cu > Hg > Pb; *Cyprinus carpio*: Cu > As > Pb > Hg and *Ictalurus furcatus:* As > Cu > Pb > Hg. For water samples: Fe > Zn > Cu > Pb > As > Hg. This order is based on the sum of geometric means for different sampling campaigns. Table 4 as a whole shows that the highest concentrations of As, Hg and Pb in fillet and gills samples occur for the Summer-Autumn sampling. Mercury shows the highest concentrations in *Ictalurus furcatus*, for gills, and in *Lepomis cyanellus* species, for fillet. The highest concentrations of arsenic for fillet and gills occur in the *Lepomis cyanellus*.

Statistical analysis was performed to search for significant differences (MANOVA *p* <0.05) for concentrations of metal(loid)s and ^238^U activity, regarding fillet and gills, the three species and the two sampling campaign. Significant differences in concentrations were found for: (1) Cu between the species and the interaction (species*tissue); (2) Fe between sampling campaigns, species, tissue and interactions (species*tissue), (tissue*sampling campaign) and (species*sampling campaign); (3) Hg between species, tissue and interaction (tissue*species); (4) Pb between sampling campaign, species and tissues; (5) Zn between tissues; (6) ^238^U between species. Appendix presents the results of the MANOVA listed above in a summary form. Tables A3 and A4 show the mean values of response functions for metal(loid)s and ^238^U activity for the biological and water samples, respectively. The tables point out for each mean value if the *p* value obtained from the significant difference is <0.05 or <0.01 (ANOVA, Tukey *post hoc* test) for the factors and their interactions, indicating if it belongs to one or another group. 

The statistical analysis for the different parameters in water samples produced the following results: the metal(loid) concentrations in water samples have not statistically significant differences (ANOVA, *p* > 0.05 ) with respect to the two sampling campaigns (Winter-Spring and Summer-Autumn); only Fe concentrations have statistically significant differences (ANOVA, *df* = 1, F = 8.29 and *p* = 0.045) between the two sampling depths (0.1 and 10 m). For water quality parameters, only two have statistically significant differences between the two sampling campaigns: TDS, (ANOVA, *df* = 1, F = 13.9 and *p* = 0.02), and temperature, (ANOVA, *df* = 1, F = 17.45 and *p* = 0.014). The detailed values of arithmetic means and *p* values are displayed in the Appendix Table A4.

**Figure 2:**
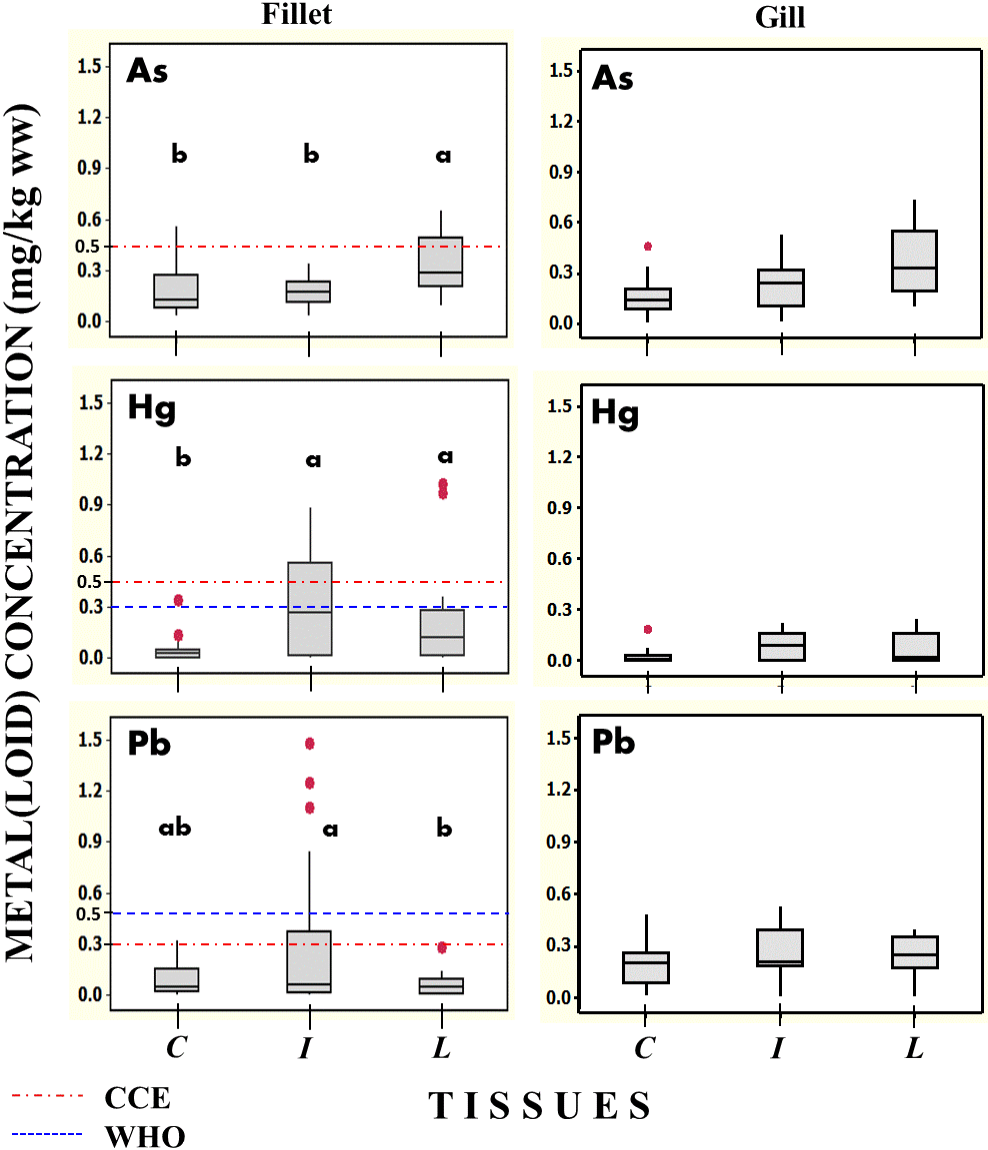
Box plot showing median values and 10th, 25 th, 75 th and 90 th percentiles of As, Hg and Pb concentrations in fillet (at left) of the three fish species analyzed (both sampling campaigns, *n* = 24). Letters C, I, L means *Cyprinus, Ictalurus, Lepomis*. Letters **a** or **b** for the species represent significant differences between the observed concentration values for the given element (*p* < 0.05), after ANOVA analysis followed by Tukey *post hoc* test. Horizontal dotted lines show the guidance levels in wet weight for human consumption reported in CCE and WHO/FAO (see text). Box plot at right shows concentrations of As, Hg and Pb in gills.

Considering together the three species studied and the two sampling campaigns, despite the observed significant differences, we can obtain an overview of the pollutants in the reservoir. Doing that, the highest concentrations of As, Hg and Pb occur in fillet and gill samples in the *Lepomis cyanellus* and *Ictalurus furcatus* species (see Figure 2)*.* In general, all metal(loid)s are found in higher concentration in gill samples, except for As and Cu in *Cyprinus carpio.* Furthermore, Hg is found in greater concentration in fillet samples.

### 3.4. Human Health Effects by Intake of Metal(loid)s and Uranium Due to Fish Fillets Consumption

Several studies about metal(loid) bioaccumulation (essential or not for the organisms) in fish have been published. Their results have shown that in some cases metal(loid)s accumulated in fish exposed to some kind of pollution (natural or anthropogenic), may jeopardize the health of the population that consume these contaminated fish [23,53,68,69]. The results of the assessment of potential health effects that may inflict the average intake of the fish species caught in the reservoir Luis L Leon are presented below.

Figure 2 shows the statistically significant differences in the concentrations of As, Hg and Pb among edible part of the three species analyzed. Furthermore, the guidance level values suggested by the Ministry of Agriculture, Fisheries and Food of the European Union (CCE) [70] are displayed. The concentration limits for Hg and Pb in fish, set by World Health Organization/Food and Agriculture Organization of United Nations (WHO/FAO) are also displayed [71].

Figure 2 presents some of the analyzed fish specimens showing As, Hg and Pb concentrations above the limit recommended by the European Union [70]. The highest concentrations of Hg and Pb occur in the species *Ictalurus furcatus.* Meanwhile, the *Lepomis cyanellus* showed the highest concentrations of As. For Cu and Zn concentrations, values exceeding the permissible limits in fillet were not observed. The details of arithmetic means and *p* values are displayed in the Appendix (Table A2).

Table 5 shows the calculated Estimated Weekly Intake (EWI) and Estimated Daily Intake (EDI), see Equation (2), according to geometric mean and maximum value of metal(loid)s concentration, in fillet species from Luis L Leon reservoir. These values were compared with the Permissible Tolerable Weekly Intake (PTWI) and Permissible Tolerable Daily Intake (PTDI). EDI were calculated in the context of essential elements required by humans for diverse metabolic activities [72]. Table 5 also shows the estimated effective dose by uranium ingestion calculated by Equation (3).

**Table 5:** Estimated theoretical annual effective dose by uranium ingestion and weekly intakes of metal(loid)s in fish fillet.

Species	*n*	Metal(loid)s Concentration *	EDI ^a^ (µg∙day^−1^)	PTDI ^b^ (µg∙day^−1^)	EWI ^c^ (µg∙week^−1^)	PTWI ^d^ (µg∙week^−1^)	H ^d^ (µSv∙year^−1^)
*Cyprinus carpio*	24	As: 0.15 (0.56)	--	--	21 (78)	350	-- -- -- -- -- -- 0.01 (0.03)
		Cu: 0.18 (0.46)	36 ** (92)	35,000	--	--	
		Fe: 4.24 (11)	85 (220)	56,000	--	--	
		Hg: 0.008 (0.34)	--	--	1.1 (48)	350	
		Pb: 0.037 (0.32)	--	--	5.2 (45)	1750	
		Zn: 11 (19)	222 (380)	71,429	--	--	
		^238^U: 0.008 (0.025)	--	--	--	--	
		^234^U: 0.012 (0.067)	--	--	--	--	
*Lepomis cyanellus*	24	As: 0.29 (0.65)	--	--	40 (91)	350	--
		Cu: 0.22 (62)	4.3 (1240)	35,000	--	--	--
		Fe: 3.24 (12)	65 (240)	56,000	--	--	--
		Hg: 0.07 (1.02)	--	--	9.7 (143)	350	--
		Pb: 0.02 (0.28)	--	--	3.2 (39)	1750	--
		Zn: 11 (18)	228 (360)	71,429	--	--	--
		^238^U: 0.012 (0.032)	--	--	--	--	--
		^234^U: 0.017 (0.041)	--	--	--	--	0.01 (0.02)
*Ictalurus furcatus*	24	As: 0.15 (0.65)	--	--	21 (91)	350	--
		Cu: 0.14 (36)	2.8 (7.2)	35,000	--	--	--
		Fe: 4.43 (12)	89 (240)	56,000	--	--	--
		Hg: 0.079 (1.02)	--	--	11 (143)	350	--
		Pb: 0.065 (0.84)	--	--	9.1 (118)	1750	--
		Zn: 9 (18)	178 (360)	71,429	--	--	--
		^238^U: 0.011 (0.034)	--	--	--	--	--
		^234^U: 0.016 (0.076)	--	--	--	--	0.01 (0.04)

EDI **^a^** = Estimated Daily Intake. Numbers in bold indicate the limit for EDI; EWI **^c^** = Estimated Weekly Intake; PTDI **^b^** = Safe daily intake level of metal(loid)s established by [73]. Numbers in bold indicate the limit for EDI; PTWI **^d^** = Safe weekly intake level of metal(loid)s [74]. Numbers in bold indicate the limit for EWI; H **^d^** = Effective dose by U ingestion; * Geometric means of metal(loid) concentrations from results in Table 3 and Table 4, in mg∙kg^−1^ ww; values in parentheses indicate the maximum value. Bold format in numbers indicates the maximum levels of metal(loid)s concentration in fish fillet (irrespective of fish species); n = sample size; ** EDI calculation (Equation (2)) for Cu in in *Cyprinus carpio* was done as follows: 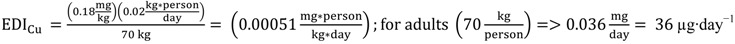.

From the results of Table 5, EWI and EDI for all metal(loid)s were far below PTWI and PTDI. From this point of view, consumption of these fish species is safe for human health. 

The annual effective dose for adults by fish consumption in this work ranged from 4.46 × 10^−3^ to 3.68 × 10^−2^ µSv∙year^−1^. This dose from uranium ingestion may be considered low when compared to the reference value equal to 0.53 µSv∙year^−1^ informed for adults in a report given by the United Nations Scientific Committee on the Effects of Atomic Radiation [57]. Also, the estimated dose for adults in this study is lower than the estimated dose for fish ingestion captured in San Marcos dam, equal to 2.23 µSv∙year^−1^ [33].

### 3.5. Discussion

**Figure A1:**
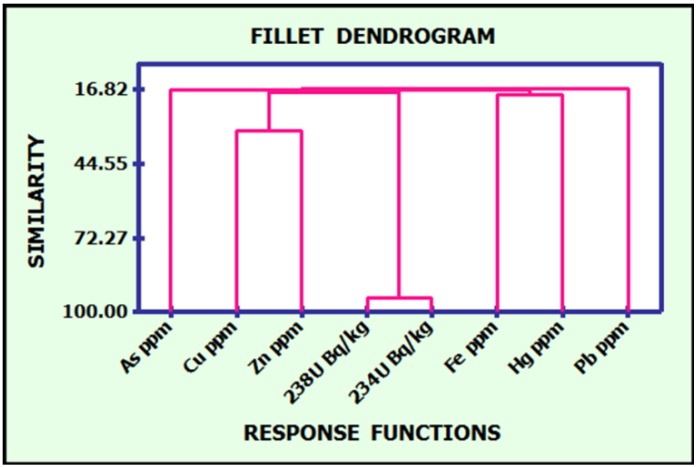
Dendrogram of similarities of response functions in fillet samples.

**Figure A2:**
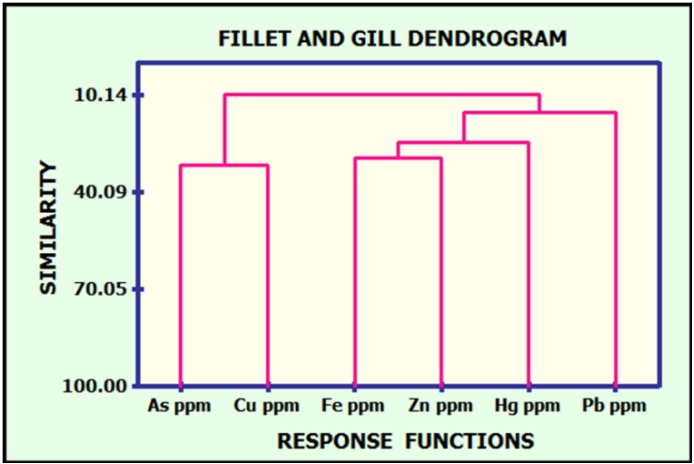
Dendrogram of similarities of response functions in fillet and gills samples.

Table 6 presents the Pearson correlation coefficients (*r*) among some sample parameters and metal(loid) concentrations in fish samples, together with their relevant p-values. Figures A1 and A2 in the Appendix present the dendrograms from significant correlations or similarities among response functions in fillets and fillets and gills, respectively. 

**Table 6:** Correlation coefficients of some parameters and metal(loid) concentrations in fillet and gill samples analyzed.

Specie	Tissue	Correlation	*n*	*r*	*p*-value
*Cyprinus carpio*	Fillet	As-Wet	24	0.500	0.013
		As-Length	24	**0.534**	**0.007**
		Cu-Fe	24	0.482	0.017
		Cu-Zn	24	**0.546**	**0.006**
	Gills	As-Wet	24	**0.525**	**0.008**
*Lepomis cyanellus*	Fillet	Hg-Wet	24	**0.563**	**0.004**
		Cu-Fe	24	**0.523**	**0.009**
		Fe-Zn	24	**0.555**	**0.005**
	Gills	Hg-Fe	24	0.402	0.052
*Ictalurus furcatus*	Gills	Fe-Pb	24	0.490	0.015
		Fe-Zn	24	0.473	0.020

Numbers in bold indicate correlation coefficients with *p*-values less than 0.01.

As Table 6 displays, *Cyprinus carpio* showed a positive correlation between arsenic concentration and fish length. *Lepomis cyanellus* presented a positive correlation between Hg concentration and fish weight. These results suggest that As and Hg concentrations tend to increase moderately as fish grows. Some authors [3,75,76] have reported a similar correlation between Hg concentration and fish length in samples of large predators like *Xiphias gladius*, *Thunnus albacares*, *Katsuwonus pelamis* and *Coryphaena hippurus*. 

Additionally, the observed metal(loid)s BAF are greater than 1. *Lepomis cyanellus* and *Ictalurus furcatus* showed the highest BAF for As, Hg and Pb, respectively. The higher concentrations of As and Hg in fillet present in *Lepomis cyanellus* specie for Summer-Autumn, may be related to the movement of this fish species towards the bottom of the water column, being in closer contact with the sediment. This may be due to conditions in the reservoir induced by higher temperatures and/or food availability. Meanwhile, *Ictalurus furcatus* has the highest concentrations of Hg and Pb when considering both sampling campaigns together. This may be due to the fact that this species remains close to sediments throughout the year. Recently, Ling *et al.* [77] have concluded that ventral muscle of tilapia, which lives in the bottom layer of ponds and reservoirs, accumulates contaminants by direct contact with sediments. On the other hand, diet of *Lepomis* and *Ictalurus* (see Table 1) includes molluscs and shellfish, which live on sediments. This conjecture is reinforced by a report of trace element concentrations in sediments along the Conchos River. It is reported that lead accumulation in sediments, given by its partition coefficient with water, is significantly higher in the Luis L Leon reservoir than in other upstream locations. Arsenic concentration value in a sediment sample from this reservoir was reported by Gutierrez *et al.* [35] as 33.0 mg∙kg^−1^in dry basis. Hernandez Garcia *et al.* [36] have reported average concentrations values of As = 9.85 mg∙kg^−1^, Cu = 3.70 mg∙kg^−1^, Pb = 63.4 mg∙kg^−1^and Zn = 79.6 mg∙kg^−1^ in sediments in dry basis from Luis L. Leon reservoir. Reported values for As and Pb concentrations in sediments of Luis L. Leon reservoir are above typical in river sediments from North America, if compared with the concentrations reported by Rice [78] in streambed sediments over the United States of America: As = 6.3 mg∙kg^−1^, Pb = 27 mg∙kg^−1^ and Hg = 0.06 mg∙kg^−1^, all given in dry basis. 

The ranges of some metal(loid) BAF in fillet samples shown in Table 5 are similar to those reported by [53] for *Sander lucioperca*, *Silurus glanis*, *Lota lota* and *Cyprinus carpio*, captured in the Danube River (Serbia) (BAF_As_ = 33.13–223.94 mg∙kg^−1^, BAF_Fe_ = 8.12–54.69 mg∙kg^−1^, BAF_Zn_ = 437.43–1879.33 mg∙kg^−1^, all given in ww). The ranges of some metal(loid) BAF in gill samples (see Table 5), are also similar to those reported by [53] (BAF_As_ = 47.6–563.64 mg∙kg^−1^, BAF_Fe_ = 98.44–186.37 mg∙kg^−1^, BAF_Zn_ = 1558.09–32,099.33 mg∙kg^−1^, ww). However, Moreno *et al.* [37] obtained higher maximum concentrations for As, Hg and Pb in both fillets and gills. The ranges of some metal(loid) BAF in the present study are higher in gill samples (except for Hg in all fish species) than in fillet samples. This feature might be explained because gills have higher bioconcentration affinity for some metal(loid)s than fillet. Thus, gills are often recommended as a better environmental indicator tissue of water pollution by metal(loid)s than fillet in fish. Type of the chemical, metabolic properties of the tissues, and the degree of environmental pollution affect the BAF levels [79].

Intake of metal(loid)s by fish is influenced by many factors such as fish species, age, sex and feeding patterns. Moreover, various environmental factors are also involved in metal(loid)s intake, such as pH, temperature, metal release into water system, physico-chemical properties of water and metal(loid)s, water depth, re-suspension processes, salinity, among other factors [58,80,81,82]. It has been established that sediments can act as sinks for a wide variety of contaminants. High concentrations of metal(loid)s in sediment causes a exposure to sediment contaminants over time, while a high concentration in water may reveal an immediate or recent source of contamination [83,84]. On the other hand, these sediments supply metals to organisms living in the close vicinity. In this context, and considering that water does not present large concentrations of metal(loid)s, as well as the diet and benthic habits of *Lepomis cyanellus* and *Ictalurus furcatus* (Table 1), high BAF for both species may be explained by the chronic exposure to some metal(loid)s such as As, Hg and Pb from sediments. 

Taking into account that contaminant concentrations increase with the weight of the specimen (Table 6), their bioaccumulation would be more dangerous for population that consume large fish. However the implications may not be affecting human health due to the low weekly intake of the target population.

## 4. Conclusions

This is the first report on uranium isotopes activity concentration in fish and water from the Luis L Leon reservoir. The activity concentration of uranium is low compared with the values reported in San Marcos dam reservoir and other mining influenced sites. This result suggests that Luis L. Leon reservoir is not affected by the transport of radionuclides, from the Victorino and San Marcos uranium deposits, or other nearby deposits. Also, annual effective dose for adults by consumptions of these fish may be considered safe, because it is below of the guidance levels values suggested by the United Nations Scientific Committee on the Effects of Atomic Radiation.

Moreover, this study provides more information about some metal(loid)s concentrations which were reported before with high values. Regarding the results of metal(loid)s, Zn and Cu are present at concentrations that are not expected to have impacts on human consumption. By contrast, comparing As, Hg and Pb concentrations in fillet with European regulations, to some extent they might pose a risk of impacts on human health. However, Estimated (Weekly or Daily) Intake values (even for the maximum values of metalloids concentration) are far below the Permissible Tolerable (Weekly or Daily) Intake levels. Moreover, almost all metal(loid) concentrations in gills showed higher values. 

The difference of metal(loid) concentrations in fillet among the studied species is likely primarily due to differences in diet and habitat. The species *Ictalurus furcatus* and *Lepomis cyanellus* show higher bioaccumulation factors of As, Hg and Pb, probably due to their benthic habitat.

## Appendix

**Table A1:** Geometric Mean values of uranium isotope AC in fish fillet (Bq∙kg^−1^ ww) and activity ratios for fish sampled at Luis L. Leon reservoir.

Sample	*n*	Tissue	Activity concentrations			
			^238^U	^234^U	U Total	^234^U/^238^U
*Cyprinus carpio* ^a^	12	Fillet	0.009 (0.003–0.025)	0.014 (0.006–0.042)	0.023 (0.009–0.067)	1.5 (1.1–1.6)
*Lepomis cyanellus* ^a^	12	Fillet	**0.014** (0.013–0.032)	**0.020** (0.009–0.039)	**0.034** (0.008–0.071)	1.4 (1.1–1.5)
*Ictalurus furcatus* ^a^	12	Fillet	0.011 (0.005–0.033)	0.017 (0.006–0.043)	0.028 (0.011–0.076)	1.4 (1.1–1.6)
*Cyprinus carpio* ^b^	12	Fillet	*0.007* (0.003–0.017)	*0.012* (0.004–0.025)	*0.017* (0.007–0.024)	1.4 (1.1–1.5)
*Lepomis cyanellus* ^b^	12	Fillet	0.011 (0.004–0.034)	0.014 (0.005–0.041)	0.025 (0.008–0.075)	1.3 (1.1–1.4)
*Ictalurus furcatus* ^b^	12	Fillet	0.011 (0.003–0.034)	0.016 (0.006–0.048)	0.027 (0.01–0.073)	1.5 (1.1–1.6)

Geometric means, values in parentheses indicate the minimum and maximum; *n* = sample size; Numbers in bold and italic formats indicate the minimum and maximum geometric mean values respectively; Letters a and b superscript after the species name indicate different sampling campaigns: ^a^ W-S = Winter-Spring, ^b^ Su-A = Summer-Autumn.

**Table A2:** Geometric mean values of metal(loid)s concentration for fillet and gills (mg∙kg^−1^ ww) in fish sampled at Luis L. Leon reservoir.

Sample	*n*	Tissue	Metal(loid)s concentration					
			As	Cu	Fe	Hg	Pb	Zn
*Cyprinus carpio* ^a^	12	Fillet	*0.13* (0.03–0.56)	0.22 (0.07–0.39)	**6** (2–11)	*0.005* (0.0003–0.05)	0.025 (0.0003–0.32)	11 (4–18)
	12	Gill	*0.11* (0.03–0.3)	0.17 (0.08–0.26)	10 (5–18)	*0.002* (0.0004–0.04)	0.132 (0.018–0.21)	37 (15–74)
*Lepomis cyanellus* ^a^	12	Fillet	0.21 (0.10–0.52)	0.18 (0.06–0.39)	*2* (1–6)	0.013 (0.0004–0.97)	*0.009* (0.0003–0.28)	9 (5–17)
	12	Gill	0.23 (0.1–0.7)	0.2 (0.01–0.3)	8.6 (5–19)	*0.009* (0.0003–0.25)	0.12 (0.011–0.38)	31 (14–64)
*Ictalurus furcatus* ^a^	12	Fillet	0.17 (0.09–0.26)	0.15 (0.08–0.28)	4 (0.3–12)	0.033 (0.0007–0.64)	0.05 (0.006–0.84)	10 (7–16)
	12	Gill	0.2 (0.07–0.43)	0.17 (0.08–0.25)	10 (6–16)	0.016 (0.0003–0.22)	*0.02* (0.19–0.39)	*28* (14–68)
*Cyprinus carpio* ^b^	12	Fillet	0.16 (0.05–0.56)	0.15 (0.02–0.46)	3 (1–8)	0.011 (0.0004–0.34)	0.05 (0.004–0.30)	11 (7–19)
	12	Gill	0.13 (0.02–0.45)	*0.14* (0.06–0.26)	**12** (8–19)	0.008 (0.0003–0.19)	0.15 (0.02–0.48)	**39** (17–85)
*Lepomis cyanellus* ^b^	12	Fillet	**0.39** (0.21–0.65)	**0.26** (0.08–0.62)	4 (1–12)	**0.126** (0.0003–1.02)	0.036 (0.0003–0.14)	**12** (4–18)
	12	Gill	**0.43** (0.15–0.68)	**0.34** (0.19–0.58)	*8* (5–15)	0.022 (0.0006–0.19)	**0.26** (0.16–0.34)	29 (15–81)
*Ictalurus furcatus* ^b^	12	Fillet	*0.13* (0.04–0.28)	*0.13* (0.01–0.36)	5 (1–12)	0.12 (0.0005–0.88)	**0.08** (0.002–1.48)	*8* (2–16)
	12	Gill	0.15 (0.01–0.52)	0.23 (0.14–0.40)	11 (6–18)	**0.039** (0.002–0.17)	0.234 (0.01–0.53)	33 (16–91)

Geometric means, values in parentheses indicate the minimum and maximum; n = sample size; Numbers in bold and italic indicate the minimum and maximum geometric mean values respectively for studied tissues and metal(loid)s; Letters a and b superscript after the species name indicate different sampling campaigns: ^a^ W-S = Winter-Spring, ^b^ Su-A = Summer-Autumn.

**Table A3:** Arithmetic means of uranium (Bq∙kg^−1^ ww) and metal(loid) concentrations (mg∙kg^−1^ ww) with probability values from factors and theirs interaction with significant differences (MANOVA, *p* < 0.05) in fish sampled at Luis L. Leon reservoir.

U/Metal(loid)s	Factor/Interaction					
	Sampling	Species	Tissue	Sampling*Species	Sampling*Tissue	Tissue*Species
^238^U	--	*C*: 0.009 (b) *I*: 0.014 (ab) *L*: 0.015 (a)	--	--	--	--
As	W-S: 0.213 (b) Su-A: 0.270 (a)	*C*: 0.180 (**b**) *I*: 0.199 (**b**) *L*: 0.347 (**a**)	--	Su-A**C*: 0.184 (**b**) Su-A**I*: 0.190 (**b**) Su-A**L*: 0.437 (**a**)	--	--
Cu	--	*C*: 0.196 (**a**) *I* : 0.187 (**a**) *L*: 0.285 (**b**)	--	--	--	F**C*: 0.255 (ab) F**I*: 0.168 (b) F**L*: 0.258 (a) G**C*: 0.167 (**b**) G**I*: 0.206 (**b**) G**L*: 0.313 (**a**)
Fe	W-S: 6.13 (b) Su-A: 7.90 (a)	*C*: 8.50 (**a**) *I*: 8.31 (**a**) *L*: 4.22 (**b**)	F: 5.06 (**b**) G: 8.96 (**a**)	W-S**C*: 8.83 (**a**) W-S**I*: 8.07 (**a**) W-S**L*: 1.48 (**b**)	Su-A*F: 4.96 (**b**) Su-A*G: 10.8 (**a**)	G**C*: 11.7 (**a**) G**I*: 10.9 (**a**) G**L*: 4.38 (**b**)
Hg	--	*C*: 0.035 (**b**) *I*: 0.197 (**a**) *L*: 0.146 (**a**)	F: 0.189 (**a**) G: 0.064 (**b**)	--	--	F**C*: 0.046 (**b**) F**I*: 0.305 (**a**) F**L*: 0.214 (**a**) G**C*: 0.025 (**b**) G**I*: 0.088 (**a**) G**L*: 0.078 (**a**)
Pb	W-S: 0.149 (b) Su-A: 0.225 (a)	*C*: 0.147 (**b**) *I*: 0.267 (**a**) *L*: 0.147 (**b**)	F: 0.143 (b) G: 0.231 (a)	--	--	--
Zn	--	--	F: 11.3 (**b**) G: 38.7 (**a**)	--	--	--

The numbers give the arithmetic mean values; a and b in parenthesis for each group indicate significant differences at *p* < 0.05 (ANOVA, Tukey *post hoc* test); Bold formats a and b in parenthesis means high significant differences at *p* < 0.01 (ANOVA, Tukey *post hoc* test); W-S = Winter-Spring, Su-A = Summer-Autumn respectively for different sampling campaigns; C = Cyprinus carpio, I = Ictalurus furcatus, L = Lepomis cyanellus respectively for different species; F = Fillet, G = Gills respectively for different tissues; ***** Represent the interaction between factors.

**Table A4:** Arithmetic means of iron concentrations, total dissolved solids and temperatures with probability values from factors and theirs interaction with significant differences (MANOVA, *p* < 0.05) in water sampled from two depth at Luis L. Leon reservoir.

Metal(loid)s/Parameters	Factor/Interaction	
	Sampling	Depth
Fe	--	0.1 m: 0.019 (b) 10 m: 0.130 (a)
TDS	W-S: 229 (a) Su-A: 131 (b)	--
T	W-S: 21 (a) Su-A: 18 (b)	--

The numbers give the arithmetic mean values; a and b in parenthesis indicate significant differences at *p* < 0.05 (ANOVA, Tukey *post hoc* test); TDS = Total Dissolved Solids T = Temperature; W-S =Winter-Spring, Su-A = Summer-Autumn, respectively, for different sampling campaigns.

## References

[1] Jabeen F., Chaudhry A. (2009). Environmental impacts of anthropogenic activities on the mineral uptake in *Oreochromis mossambicus* from indus river in Pakistan. Environ. Monit. Assess..

[2] Quintero-Alvarez J.M., Soto-Jiménez M.F., Amezcua F., Voltolina D., Frías-Espericueta M. (2012). Cadmium and lead concentrations in the fish tissues of a coastal lagoon system of the Gulf of California. Bull. Environ. Contam. Toxicol..

[3] Ouédraogo O., Amyot M. (2012). Mercury, arsenic and selenium concentrations in water and fish from sub-Saharan semi-arid freshwater reservoirs (Burkina Faso). Sci. Total Environ..

[4] Türkmen M., Türkmen A., Tepe Y., Ates A., Gökkus K. (2008). Determination of metal contaminations in sea foods from Marmara, Aegean and Mediterranean seas: Twelve fish species. Food Chem..

[5] Korkmaz Görür F., Keser R., Akçay N., Dizman S. (2012). Radioactivity and heavy metal concentrations of some commercial fish species consumed in the Black Sea region of Turkey. Chemosfere.

[6] Labrot F., Narbonne J.F., Ville P., Saint Denis M., Ribera D. (1999). Acute toxicity, toxicokinetics, and tissue target of lead and uranium in the clam *Corbicula fluminea* and the worm *Eisenia fetida*: Comparison with the fish *Brachydanio rerio*. Arch. Environ. Contam. Toxicol..

[7] Schenone N.F., Avigliano E., Goessler W., Fernández Cirelli A. (2014). Toxic metals, trace and major elements determined by ICPMS in tissues of *Parapimelodus valenciennis* and *Prochilodus lineatus* from Chascomus lake, Argentina. Microchem. J..

[8] WHO, World Health Organization Arsenic, Fact Sheet No. 372, (2012). http://www.who.int/mediacentre/factsheets/fs372/en/#.09/27/2013.

[9] WHO, World Health Organization Lead Poisoning and Health, Fact Sheet No. 379, (2013). http://www.who.int/mediacentre/factsheets/fs379/en/.

[10] Domingo J.L. (2001). Reproductive and developmental toxicity of natural and depleted uranium: A review. Reprod. Toxicol..

[11] Al Sayegh Petkovsek S., Mazej Grudnik Z., Pokorny B. (2012). Heavy metals and arsenic concentrations in ten fish species from the Salek lakes (Slovenia): Assessment of potential human health risk due to fish consumption. Environ. Monit. Assess..

[12] Burger J., Gaines K., Boring C.S., Stephens W.L., Snodgrass J., Dixon C., McMahon M., Shukla S., Shukla T., Gochfeld M. (2002). Metal levels in fish from the Savannah river: Potential hazards to fish and other receptors. Environ. Res..

[13] Nawaz S., Nagra S.A., Saleem Y., Priyadarshi A. (2010). Determination of heavy metals in fresh water fish species of the River Ravi, Pakistan compared to farmed fish varieties. Environ. Monit. Assess..

[14] Wang X., Sato T., Xing B., Tao S. (2005). Health risks of heavy metals to the general public in Tianjin, China via consumption of vegetables and fish. Sci. Total Environ..

[15] Franco J.L., Posser T., Mattos J.J., Sánchez-Chardi A., Trevisan R., Oliveira C.S., Carvalho P.S.M., Leal R.B., Marques M.R.F., Bainy A.C.D. (2008). Biochemical alterations in juvenile carp (*Cyprinus carpio*) exposed to zinc: Glutathione reductase as a target. Mar. Environ. Res..

[16] Luna Porres M.Y., Montero Cabrera M.E., Manjón Collado G., Díaz Frances I., Rentería Villalobos M. (2012). Determination of uranium and polonium in *Sparus aurata* by alpha spectrometry. Revista Mexicana de Física.

[17] Castro-González M.I., Méndez-Armenta M. (2008). Heavy metals: Implications associated to fish consumption. Environ. Toxicol. Pharmacol..

[18] Cyrille Y.D.A., Victor K., Sanogo T.A., Boukary S., Joseph W. (2012). Cadmium accumulation in tissues of *Sarotherodon melanotheron* (Rüppel, 1852) from the Aby lagoon system in Côte D’ivoire. Int. J. Environ. Res. Public Health.

[19] Buet A., Barillet S., Camilleri V. (2005). Changes in oxidative stress parameters in fish as response to direct uranium exposure. Radioprotection.

[20] Beatrice G., Isabelle C., Virginie C., Christelle A.-G. (2013). Effects of depleted uranium on oxidative stress, detoxification, and defence parameters of Zebrafish *Danio rerio*. Arch. Environ. Contam. Toxicol..

[21] Díaz-Francés I., Mantero J., Manjón G., Díaz J., García-Tenorio R. (2013). ^210^po and ^238^u isotope concentrations in commercial bottled mineral water samples in Spain and their dose contribution. Radiat. Prot. Dosim..

[22] Stromman G., Rosseland B.O., Skipperud L., Burkitbaev L.M., Uralbekov B., Heier L.S., Salbu B. (2013). Uranium activity ratio in water and fish from Pit lakes in Kurday, Kazakhstan and Taboshar, Tajikistan. J. Environ. Radioact..

[23] Skipperud L., Stromman G., Yunusov M., Stegnar P., Uralbekov B., Tilloboev H., Zjazjev G., Heier L.S., Rosseland B.O., Salbu B. (2013). Environmental impact assessment of radionuclide and metal contamination at the former U sites Taboshar and Digmai, Tajikistan. J. Environ. Radioact..

[24] Skipperud L., Jørgensen A.G., Heier L.S., Salbu B., Rosseland B.O. (2013). Po-210 and Pb-210 in water and fish from Taboshar uranium mining Pit lake, Tajikistan. J. Environ. Radioact..

[25] Yılmaz F., Özdemir N., Demirak A., Tuna A.L. (2007). Heavy metal levels in two fish species *Leuciscus cephalus* and *Lepomis gibbosus*. Food Chem..

[26] Alhas E., Oymak S.A., Karadede Akin H. (2009). Heavy metal concentrations in two barb, *Barbus xanthopterus* and *Barbus rajanorum* mystaceus from Ataturk dam lake, Turkey. Environ. Monit. Assess..

[27] Salbu B., Skipperud L. (2007). Challenges in Radioecotoxicology. Multiple Stressors: A Challenge for the Future.

[28] Salbu B. (2007). Speciation of radionuclides–analytical challenges within environmental impact and risk assessments. J. Environ. Radioact..

[29] Sandoval-Solis S. (2011). Water Planning and Management for Large Scale River Basins: Case of Study of the Rio Grande/Rio Bravo Transboundary Basin.

[30] Ferríz H. (1985). Uranium mineralization in the san marcos volcanic center Chihuahua, Mexico. Procedings of the Technical Committee Meeting, Uranium Deposits in Volcanic Rocks.

[31] Colmenero Sujo L., Montero Cabrera M.E., Villalba L., Rentería Villalobos M., Torres Moye E., García León M., García-Tenorio R., Mireles García F., Herrera Peraza E.F., Sánchez Aroche D. (2004). Uranium-238 and thorium-232 series concentrations in soil, radon-222 indoor and drinking water concentrations and dose assessment in the city of Aldama, Chihuahua, Mexico. J. Environ. Radioact..

[32] Luna Porres M.Y., Alarcon Herrera M.T., Montero Cabrera M.E., Rodriguez Villa M.A., Villalobos M.R., Peraza E.H. (2011). *Baccharis salicifolia* development in the presence of high concentrations of uranium in the arid environment of San Marcos, Chihuahua. Revista Mexicana de Física.

[33] Renteria-Villalobos M., Cortes M., Mantero J., Manjon G., Garcia-Tenoria R., Herrera E., Montero-Cabrera M. (2012). Uranium in the surrounding of San Marcos-Sacramento river environment (Chihuahua, Mexico). Sci. World J..

[34] De la Mora-Covarrubias A., Quiñonez-Martinez M., Sosa-Cerecedo M., Soto-Cruz R.  Estudio de la calidad del agua del Río Bravo en el área de influencia de Cd. Juárez, Chihuahua-El Paso, Texas. Proceeding of the VI Congreso Internacional y XII Nacional de Ciencias Ambientales.

[35] Gutierrez M., Alarcón-Herrera M., Camacho L. (2009). Geographical distribution of arsenic in sediments within the rio Conchos basin, Mexico. Environ. Geol..

[36] Hernández-Garcia Y., Sosa-Cerecedo M., Moreno M., Alcalá J., Puga S. (2008). Evaluación de la contaminación por metales pesados y arsénico en sedimento en embalses del estado de Chihuahua, México. Revista Latinoamericana de Recursos Naturales.

[37] Moreno-López M.V., Sosa M., Patiño R., Benavides A., Miranda S.V., Rubio A.D., Leal L. Accumulation of Arsenic and Mercury in Mojarra, Catfish and Carp Fish Species from Three Water Reservoirs in Chihuahua State. Proceedings of the 10th International Conference on the Biogeochemistry of Trace Elements.

[38] INEGI (1999). Estudio hidrológico del estado de Chihuahua.

[39] L’Annunziata M.F., Kessler M.J. (2003). Liquid Scintillation Analysis: Principles and Practice. Handbook of Radioactivity Analysis.

[40] SCFI (2001). NMX-AA-051-SCFI-2001, Water Analysis—Determination of Metals by Atomic Absorption in Natural, Drinking, Wastewaters and Treated Wastewaters—Test Method.

[41] HACH (2008). Water Analysis Handbook.

[42] SCFI (2001). NMX-AA-073-SCFI-2001, Water Analysis—Determination of Total Chlorine in Natural, Drinking, Wastewaters and Treated Wastewaters—Test Method.

[43] SCFI (2001). NMX-AA-036-SCFI-2001, Water Analysis—Determination of Acidity and Total Alkalinity in Natural, Drinking, Wastewaters and Treated Wastewaters.

[44] Blanco Rodriguez M.P., Vera Tomé F., Lozano J.C., Gómez Escobar V. (2000). Sequential method for the determination of uranium, thorium and ^226^Ra by liquid scintillation alpha spectrometry. Appl. Radiat. Isot..

[45] Currie L.A. (1968). Limits for qualitative detection and quantitative determination. Application to radiochemistry. Anal. Chem..

[46] Jabeen F., Chaudhry A. (2009). Monitoring trace metals in different tissues of *Cyprinus carpio* from the Indus river in Pakistan. Environ. Monitor. Assess..

[47] Scerbo R., Ristori T., Stefanini B., de Ranieri S., Barghigiani C. (2005). Mercury assessment and evaluation of its impact on fish in the Cecina river basin (Tuscany, Italy). Environ. Pollut..

[48] Karadede H., Oymak S.A., Ünlü E. (2004). Heavy metals in mullet, liza abu, and catfish, *Silurus triostegus*, from the Atatürk dam lake (Euphrates), Turkey. Environ. Int..

[49] Lau S., Mohamed M., Yen A.T., Su’ut S. (1998). Accumulation of heavy metals in freshwater molluscs. Sci. Total Environ..

[50] Shah A.Q., Kazi T.G., Arain M.B., Jamali M.K., Afridi H.I., Jalbani N., Baig J.A., Kandhro G.A. (2009). Accumulation of arsenic in different fresh water fish species—Potential contribution to high arsenic intakes. Food Chem..

[51] Zhang N., Wei C., Yang L. (2013). Occurrence of arsenic in two large shallow freshwater lakes in China and a comparison to other lakes around the world. Microchem. J..

[52] Weisbrod A.V., Burkhard L.P., Arnot J., Mekenyan O., Howard P.H., Russom C., Boethling R., Sakuratani Y., Traas T., Bridges T. (2007). Workgroup report: Review of fish bioaccumulation databases used to identify persistent, bioaccumulative, toxic substances. Environ. Health Perspect..

[53] Subotic S., Spasic S., Visnjic-Jeftic Z., Hegedis A., Krpo-Cetkovic J., Mickovic B., Skoric S., Lenhardt M. (2013). Heavy metal and trace element bioaccumulation in target tissues of four edible fish species from the Danube river (Serbia). Ecotoxicol. Environ. Saf..

[54] Food and Agriculture Organization (FAO) (2005). Statistics Division, Food Security Statistics, Food Consumption.

[55] Zhuang P., McBride M.B., Xia H., Li N., Li Z. (2009). Health risk from heavy metals via consumption of food crops in the vicinity of dabaoshan mine, south China. Sci. Total Environ..

[56] International Commission on Radiological Protection (ICRP) (1990). Recommendations of the International Commission on Radiological Protection.

[57] UNSCEAR, United Nation Scientific Committee on the Effects of Atomic Radiation (2008). Exposures from natural radiation sources (Annex B).

[58] Carvalho F.P., Oliveira J.M., Lopes I., Batista A. (2007). Radionuclides from past ur anium mining in rivers of Portugal. J. Environ. Radioact..

[59] Kraemer L.D., Evans D. (2012). Uranium bioaccumulation in a freshwater ecosystem: Impact of feeding ecology. Aquat. Toxicol..

[60] Burillo Montúfar J.C., Reyes Cortés M., Reyes Cortés I.A., Espino Valdez M.S., Hinojosa de la Garza O.R., Nevárez Ronquillo D.P., Herrera Peraza E., Rentería Villalobos M., Montero Cabrera M.E. (2012). Uranium-series isotopes transport in surface, vadose and ground waters at San Marcos uranium bearing basin, Chihuahua, Mexico. Appl. Geochem..

[61] Reyes-Cortés M., Reyes-Cortés I.A., Espino Valdez S., Rentería-Villalobos M., Burillo Montúfar J.C., Montero-Cabrera M.E.  (2012). Origen y distribución de la radiactividad natural en la zona norte de la cuenca de Chihuahua, México. Revista Mexicana de Ciencias Geológicas.

[62] Panorama Minero del Estado de Chihuahua. http://www.sgm.gob.mx/pdfs/CHIHUAHUA.pdf.

[63] De la Maza Benignos M. (2009). Los peces del Río Conchos.

[64] Al-Masri M.S., Mamish S., Budeir Y., Nashwati A. (2000). 210po and 210pb concentrations in fish consumed in Syria. J. Environ. Radioact..

[65] Bustamante P., Germain P., Leclerc G., Miramand P. (2002). Concentration and distribution of 210po in the tissues of the scallop *Chlamys varia* and the mussel *Mytilus edulis* from the coasts of Charente-Maritime (France). Mar. Pollut. Bull..

[66] CNA, Comisión Nacional del Agua (2001). Las represas del estado de Chihuahua.

[67] Gutiérrez M., Borrego P. (1999). Water quality assessment of the rio Conchos, Chihuahua, Mexico. Environ. Int..

[68] Turkmen M., Turkmen A., Tepe Y. (2011). Comparison of metals in tissues of fish from Paradeniz lagoon in the coastal area of northern east Mediterranean. Bull. Environ. Contam. Toxicol..

[69] Otter R.R., Bailey F.C., Fortner A.M., Adams S.M. (2012). Trophic status and metal bioaccumulation differences in multiple fish species exposed to coal ash-associated metals. Ecotoxicol. Environ. Saf..

[70] Portant Fixation de Teneurs Maximales Pour Certains Contaminants dans les Denrées Alimentaires. http://eur-lex.europa.eu/legal-content/FR/TXT/PDF/?uri=CELEX:32006R1881&from=FR.

[71] WHO/FAO, World Health Organization, Food and Agriculture Organization of the United Nations (1989). Evaluation of Certain Food Additives and the Contaminants Mercury, Lead and Cadmium.

[72] Patra A.K., Wagh S.S., Jain A.K., Hegde A.G. (2010). Assessment of daily intake of trace elements by Kakrapar adult population through ingestion pathway. Environ. Monit. Assess..

[73] JECFA (2010). Evaluation of Certain Food Additives and Contaminants: Seventy Second Report of the Joint FAO/WHO Expert Committe on Food Additives.

[74] FAO/WHO, Food and Agriculture Organization of the United Nations and World Health Organization (2004). Summary of Evaluations Performed by the Joint FAO/WHO Expert Committe on Food Additives (JECFA 1956–2003).

[75] Adams D.H. (2009). Consistently low mercury concentrations in dolphinfish, *Coryphaena hippurus*, an oceanic pelagic predator. Environ. Res..

[76] Kojadinovic J., Potier M., Le Corre M., Cosson R.P., Bustamante P. (2007). Bioaccumulation of trace elements in pelagic fish from the western Indian ocean. Environ. Pollut..

[77] Ling M.-P., Wu C.-C., Yang K.-R., Hsu H.-T. (2013). Differential accumulation of trace elements in ventral and dorsal muscle tissues in tilapia and milkfish with different feeding habits from the same cultured fishery pond. Ecotoxicol. Environ. Saf..

[78] Rice K. (1999). Trace-element concentrations in streambed sediment across the conterminous United States. Environ. Sci. Technol..

[79] Uysal K., Köse E., Bülbül M., Dönmez M., Erdoğan Y., Koyun M., Ömeroğlu Ç., Özmal F. (2009). The comparison of heavy metal accumulation ratios of some fish species in Enne Dame lake (Kütahya/Turkey). Environ. Monitor. Assess..

[80] Kanayochukwu N.J., Ebere O.O., Obi O.I. (2010). Nigeria: Environmental health concerns. Encycl. Environ. Health.

[81] Rejomon G., Nair M., Joseph T. (2010). Trace metal dynamics in fishes from the southwest coast of India. Environ. Monit. Assess..

[82] Campbell L., Verburg P., Dixon D.G., Hecky R.E. (2008). Mercury biomagnification in the food web of lake Tanganyika (Tanzania, east Africa). Sci. Total Environ..

[83] Karadede-Akin H., Ünlü E. (2007). Heavy metal concentrations in water, sediment, fish and some benthic organisms from Tigris River, Turkey. Environ. Monitor. Assess..

[84] Baldantoni D., Maisto G., Bartoli G., Alfani A. (2005). Analyses of three native aquatic plant species to assess spatial gradients of lake trace element contamination. Aquat. Bot..

